# All-cause mortality among Danish nursing home residents before and during the COVID-19 pandemic: a nationwide cohort study

**DOI:** 10.1007/s10654-023-00994-6

**Published:** 2023-04-03

**Authors:** Mikkel Porsborg Andersen, Elisabeth Helen Anna Mills, Alessandra Meddis, Kathrine Kold Sørensen, Jawad Haider Butt, Lars Køber, Henrik Enghusen Poulsen, Matthew Phelps, Gunnar Gislason, Helle Collatz Christensen, Morten Schou, Emil L. Fosbøl, Thomas Alexander Gerds, Kristian Kragholm, Christian Torp-Pedersen

**Affiliations:** 1 Department of Cardiology, Nordsjællands Hospital, Dyrehavevej 29, 3400 Hillerød, Denmark; 2 Department of Cardiology, Aalborg University Hospital, Aalborg, Denmark; 3 Department of Biostatistics, University of Copenhagen, Copenhagen, Denmark; 4 Department of Cardiology, Rigshospitalet, Copenhagen, Denmark; 5 Department of Endocrinology, Copenhagen University Hospital at Bispebjerg Frederiksberg Hospital, Copenhagen, Denmark; 6 The Danish Heart Foundation, Copenhagen, Denmark; 7 Department of Cardiology, Herlev-Gentofte Hospital, Copenhagen, Denmark; 8 Department of Clinical Medicine, University of Copenhagen, Copenhagen, Denmark; 9 National Institute of Public Health, University of Southern Denmark, Copenhagen, Denmark; 10 Copenhagen Emergency Medical Services, Copenhagen, Denmark; 11 Unit of Clinical Biostatistics and Epidemiology, Aalborg University Hospital, Aalborg, Denmark

**Keywords:** Nursing home residents, Care home facilities, All-cause mortality, SARS-CoV-2, COVID-19

## Abstract

A substantial part of mortality during the COVID-19-pandemic occurred among nursing home residents which caused alarm in many countries. We investigate nursing home mortality in relation to the expected mortality prior to the pandemic. This nationwide register-based study included all 135,501 Danish nursing home residents between 2015 until October 6, 2021. All-cause mortality rates were calculated using a standardization method on sex and age distribution of 2020. Survival probability and lifetime lost for 180 days was calculated using Kaplan Meier estimates. Of 3,587 COVID-19 related deaths, 1137 (32%) occurred among nursing home residents. The yearly all-cause mortality rates per 100,000 person-years in 2015, 2016, and 2017 were 35,301 (95% CI: 34,671–35,943), 34,801 (95% CI: 34,180–35,432), and 35,708 (95% CI: 35,085–36,343), respectively. Slightly elevated mortality rates per 100,000 person-years were seen in 2018, 2019, 2020, and 2021 of 38,268 (95% CI: 37,620–38,929), 36,956 (95% CI: 36,323–37,600), 37,475 (95% CI: 36,838–38,122), and 38,536 (95% CI: 37,798–39,287), respectively. For SARS-CoV-2-infected nursing home residents, lifetime lost difference was 42 days (95% CI: 38–46) in 2020 versus non-infected in 2018. Among vaccinated in 2021, lifetime lost difference was 25 days (95% CI: 18–32) for SARS-CoV-2-infected versus non-infected. Even though a high proportion of COVID-19 fatalities took place in nursing homes and SARS-CoV-2-infection increased the risk of individual death, the annual mortality was only slightly elevated. For future epidemics or pandemics reporting numbers of fatal cases in relation to expected mortality is critical.

**Supplementary Information:**

The online version contains supplementary material available at 10.1007/s10654-023-00994-6.

## Introduction

During the corona virus disease (COVID-19) pandemic there has been a major focus on the mortality related to severe acute respiratory syndrome coronavirus 2 (SARS-CoV-2) infection with numbers of deaths being the major communicated finding. While such numbers indicate a serious situation, they ignore the fact that all countries have high numbers of people dying every year. This situation is particularly relevant in nursing homes where the mortality has been high during the pandemic. A recently published study by Thompson et al. concluded that between 19 and 72% of all COVID-19-related deaths occurred within nursing home facilities in the following countries: Australia, Belgium, Canada, France, Germany, Spain, United States, England, and Wales [1]. As of January 25th, 2022, The Danish Health Authority reported a total of 3587 COVID-19 related deaths in Denmark of which 1137 (32%) occurred among nursing home residents [2]. Nursing homes are often the last permanent place of residence for the most frail and comorbid elderly with short life expectancy, as nursing home residents often suffer from severe health problems and immobility [1, 3–6], which are also factors that have shown to contribute to severe outcomes and deaths in relation to COVID-19 [4, 5, 7]. It is therefore important to set the number of people dying in connection with COVID-19 in relation to the life expectancy of nursing home residents. Among nursing home residents, the total number of fatal cases can potentially create a false impression of excess mortality if the life expectancy of those infected is already very short. In order to examine the mortality numbers from the perspective of background life expectancy, the present study aims to compare mortality in Danish nursing homes during the 2020/2021 COVID-19 pandemic with mortality during the pre-pandemic years of 2015–2019, with particular emphasis on a comparison to 2018, a year where the influenza vaccine failed to deliver protection [8]. Finally, this study also examined the impact of SARS-CoV-2 infection on mortality among nursing home residents, with emphasis on obtaining lifetime difference of SARS-CoV-2-infection in 2020 compared to the pre-pandemic years of 2015–2019 as well as the lifetime difference among vaccinated nursing home residents for SARS-CoV-2-infected versus non-infected in 2021.

## Methods

### Study setting, population, and design

This nationwide register-based cohort study comprised all nursing home residents in Denmark from January 1, 2015, to the last date of data availability on October 6, 2021. In this study, nursing home residents are defined as individuals who have been living at a valid nursing home address and were between the ages of 50 and 102 within the study period. The upper age limit of 102 year was chosen as only 14 persons was above this age and these were necessary to exclude as it was not possible to identify complete matches. The information on nursing homes was obtained from the Danish Health Data Authority, which administrates a full address list of all Danish nursing homes since 2014 [9]. Through both the Danish Health Data Authority and Statistics Denmark, it was possible to obtain civil personal registration number of each nursing home resident living at a nursing home address.

### Data sources

In Denmark, each resident is assigned a unique civil personal registration number at birth or upon immigration. This identification is used in national administrative registries for economic, social, and healthcare purposes and enables linkage between all Danish administrative registries. From the Danish Nursing Home register, the following information was obtained: nursing home address, validity dates, moving-in and -out dates of nursing home residents [9]. Information on date of birth, sex, and date of death was obtained from the Danish Civil Registration System [10]. Information on SARS-CoV-2 test results and date of tests was obtained from the Danish Microbiology Database, which automatically receives test results from the country's microbiology departments [11, 12]. Information on all hospitalizations, related diagnoses, and dates was obtained from the Danish National Patient Register [13]. Information on redeemed medication prescriptions was obtained from the Danish National Prescription Register [14]. Information on dates of first and second vaccines was obtained from the Danish Vaccination Register [15]. Lastly, information on date of death was also obtained from the Danish Cause of Death Register when available [16].

### Covariates

Chronic conditions were based on all-time primary- and secondary-diagnoses registered in the Danish National Patient Register and coded according to the International Classification of Disease, Eighth and Tenth Revision (ICD-8 and ICD-10) [17]. The chronic conditions included were cardiac diseases, other circulatory diseases, all types of cancer except non-melanoma skin cancer, chronic airways diseases (chronic obstructive pulmonary diseases, asthma, and interstitial lung diseases), diabetes, chronic kidney disease, Alzheimer’s, and dementia; for further details see supplementary Table S1 in supplemental appendix. Cardiovascular disease was assessed as a combination of cardiac diseases and other circulatory diseases. Nursing home residents were also classified as having diabetes if they ever had a redeemed prescription for an antidiabetic drug (ATC-code; A10). Likewise, residents were classified as having hypertension if they had a minimum of two redeemed prescriptions for antihypertensive drugs within 180 days prior to January 1 each year. All chronic conditions above except cancer were considered permanent, and only cancer diagnoses within 10 years or less prior to January 1 each year were considered permanent, in accordance with previously published study [18]. Nursing home residents’ age and sex were retrieved from the Danish Civil Registration System. In the calculation, weeks was classified as starting on January 1 of each year from 2015 through to 2021. The SARS-CoV-2-infection status of nursing home residents was retrieved from the Danish Microbiology Database [11, 12] and classified into two categories, negative and positive polymerase chain reaction tests for SARS-CoV-2, as well as according to the date when the tests were taken. The vaccine status of the nursing home residents was assessed through dates of first and second vaccine [15]. Nursing homes residents were defined as fully vaccinated on the date the resident has received both vaccine doses of the same type of vaccine.

### Outcomes

The primary outcome was all-cause mortality. Secondary outcomes were lifetime lost due to the SARS-CoV-2-infection in 2020, compared to the pre-pandemic years of 2015 to 2019, and lifetime lost due to SARS-CoV-2-infection among vaccinated nursing home residents in 2021.

### Statistical analysis

To calculate the weekly mortality rates, the number of deaths in the nursing homes was divided by the number of person days in nursing homes in that week. We used the age distribution (1-year intervals) and sex distribution of all nursing home residents on January 1, 2020, as reference in the calculations. The standardized weekly mortality rates, as number of events per 100,000 person-weeks, were reported for each year from 2015 to October 6, 2021. We performed a sensitivity analysis starting time in week 40 to investigate seasonal infectious disease variation. The standardized yearly mortality rates with 95% confidence intervals (95% CI) were likewise reported for each year as number of events per 100,000 person-years using the same reference distribution, using the Gamma method [19]. For all SARS-CoV-2-infected residents in 2020, the index date was set at the date of positive SARS-CoV-2 test. Each infected resident (i.e., case) was matched with 5 nursing home residents (i.e., controls) from 2018 who had the same sex and age. The index date for the controls was set 2 years before the case’s index date. In the supplementary appendix analysis, the same matching approach was used, but instead controls were selected from the other pre-pandemic years (i.e., 2015, 2016, 2017, and 2019). Similarly, we considered all first breakthrough SARS-CoV-2 cases in 2021, i.e., SARS-CoV-2-infection that occurred after the 2nd vaccination date and set the index date at the date of infection. For each case, 5 controls were matched from the vaccinated nursing home population in 2021 who did not yet have SARS-CoV-2 at the case index date. Separately for case/control groups and according to sex, we estimated the survival probability curve after the index date using the Kaplan–Meier method. The restricted mean lifetime was calculated as the area under the survival curve up to 180 days after the index date. The lifetime lost was calculated as the difference between 180 days and the restricted mean lifetime [20]. We report the difference between the lifetime lost in days among cases versus matched controls with 95% CI using the plug-in estimator [21]. We performed sensitivity analyses extending the area under the survival curve up to 365 days after the index date to test if this altered the results. Data management and analyses were performed using R statistical software version 4.0.3 [22].

### Ethical approval

In Denmark, register-based studies that are performed for the sole purpose of statistics and scientific research do not require ethical committee approval or patient consent, in accordance with The Danish Data Protection Act [23]. Approval to use the data sources for research purposes was granted by the data responsible institute in the Capital Region of Denmark (approval number P-2019-191), in accordance with the General Data Protection Regulation (GDPR). Data are accessed on secure servers under Statistics Denmark and cannot be shared according to Danish legislation.

## Results

A total of 136,749 residents who had a nursing home address registered in the Danish Nursing Home register within the study period were identified. Of these, 1248 were excluded for being outside the age range, leaving a study population of 135,501 nursing home residents. In total 92,870 of the nursing home residents died within the study period. The number of nursing home residents was consistent across the years, with the majority being female (Table 1). There were minor variations in the distribution of comorbidities, and almost 88% of the residents had at least one of the presented comorbidities. The total number of COVID-19-infected residents in 2020 was 2340 and 1719 were infected in 2021 until the end of the study period on October 6.

**Table 1:** Characteristics of the 135,501 nursing home residents included in study

Variables	Years							Total
	2015	2016	2017	2018	2019	2020	2021	
Nursing home residents within each year [No.]*	53,549	54,077	54,684	55,608	55,131	55,001	51,819	379,869
Residents’ entries at Nursing Home within each year [No.]	13,491	13,732	13,856	14,662	14,111	14,064	11,274	95,190
Mortality within each year [No.]	12,985	13,013	13,437	14,298	13,919	14,140	11,078	92,870
Sex – Male [No. (%)]	18,399 (34.4)	18,911 (35.0)	19,405 (35.5)	20,109 (36.2)	20,129 (36.5)	20,387 (37.1)	19,146 (36.9)	136,486 (35.9)
Age on January 1 of each year Median [iqr]	85.1 [78.0–90.3]	85 [77.8–90.3]	84.9 [77.8–90.3]	84.7 [77.6–90.3]	84.6 [77.5–90.3]	84.5 [77.4–90.1]	84.5 [77.5–90.1]	84.8 [77.6–90.2]
Chronic Pulmonary Disease [No. (%)]	8259 (15.4)	8521 (15.8)	8837 (16.2)	9107 (16.4)	9059 (16.4)	8971 (16.3)	8600 (16.6)	61,354 (16.2)
Chronic Kidney Disease [No. (%)]	2577 (4.8)	2750 (5.1)	2917 (5.3)	2971 (5.3)	2991 (5.4)	3068 (5·6)	2885 (5.6)	20,159 (5.3)
Diabetes [No. (%)]	9865 (18.4)	9876 (18.3)	9955 (18.2)	10,032 (18.0)	9641 (17.5)	9322 (16.9)	8489 (16.4)	67,180 (17.7)
Total Cardiovascular Disease [No. (%)]	37,719 (70.4)	38,132 (70.5)	38,469 (70.3)	38,923 (70.0)	38,217 (6.3)	37,486 (68.2)	35,110 (67.8)	264,056 (69.5)
Total Cardiac [No. (%)]	10,282 (19.2)	10,257 (19.0)	10,285 (18.8)	10,235 (18.4)	9948 (18.0)	9723 (17.7)	9088 (17.5)	69,818 (18.4)
Heart failure [No. (%)]	7078 (13.2)	7074 (13.1)	7128 (13.0)	7146 (12.9)	6870 (12.5)	6739 (12.3)	6319 (12.2)	48,354 (12.7)
Myocardial Infarction [No. (%)]	5070 (9.5)	5034 (9.3)	5073 (9.3)	4969 (8.9)	4837 (8.8)	4692 (8.5)	4362 (8.4)	34,037 (9.0)
Total Other Circulatory Disease [No. (%)]	36,085 (67.4)	36,520 (67.5)	36,842 (67.4)	37,247 (67.0)	36,515 (66.2)	35,742 (65.0)	33,476 (64.6)	252,427 (66.5)
Hypertension [No. (%)]	29,205 (54.5)	29,671 (54.9)	29,980 (54.8)	30,256 (54.4)	29,554 (53.6)	28,607 (52.0)	26,467 (51.1)	203,740 (53.6)
Peripheral Vascular Disease [No. (%)]	5340 (10.0)	5518 (10.2)	5638 (10.3)	5743 (10.3)	5672 (10.3)	5502 (10.0)	5098 (9.8)	38,511 (10.1)
Cerebrovascular Disease [No. (%)]	18,312 (34.2)	18,403 (34.0)	18,575 (34.0)	18,908 (34.0)	18,610 (33.8)	18,422 (33.5)	17,485 (33.7)	128,715 (33.9)
Cancer (last diagnosis code ≤ 10 years ago) [No. (%)]	8061 (15.1)	8241 (15.2)	8184 (15.0)	8232 (14.8)	8026 (14.6)	7734 (14.1)	6794 (13.1)	55,272 (14.6)
Dementia [No. (%)]	19,329 (36.1)	19,873 (36.7)	20,105 (36.8)	20,541 (36.9)	20,430 (37.1)	20,495 (37.3)	19,123 (36.9)	139,896 (36.8)
None of the above [No. (%)]	6021 (11.2)	6045 (11.2)	6164 (11.3)	6286 (11.3)	6303 (11.4)	6599 (12.0)	6443 (12.4)	43,861 (11.5)

Interquartile range [iqr], Number [No.]

*Represent how many of the 135,501 nursing home residents that are living in a nursing home within each year for the study period, residents can appear multiple times

### Primary outcome

**Fig. 1:**
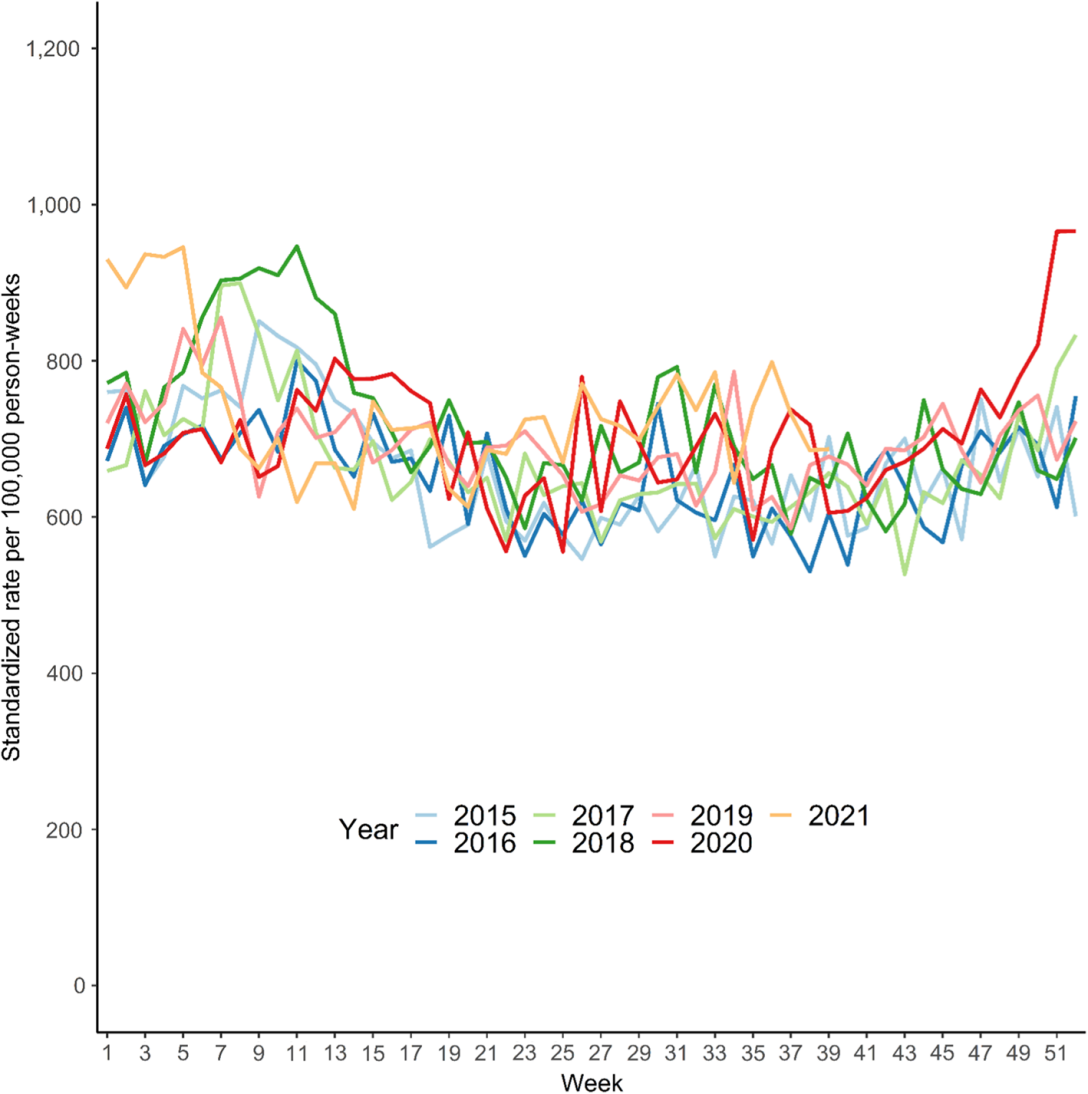
Age- and sex-standardized weekly all-cause mortality rates as number of events per 100,000 person-weeks starting on January 1 for each year from 2015 until October 6, 2021, among 135,501 Danish nursing home residents

**Fig. 2:**
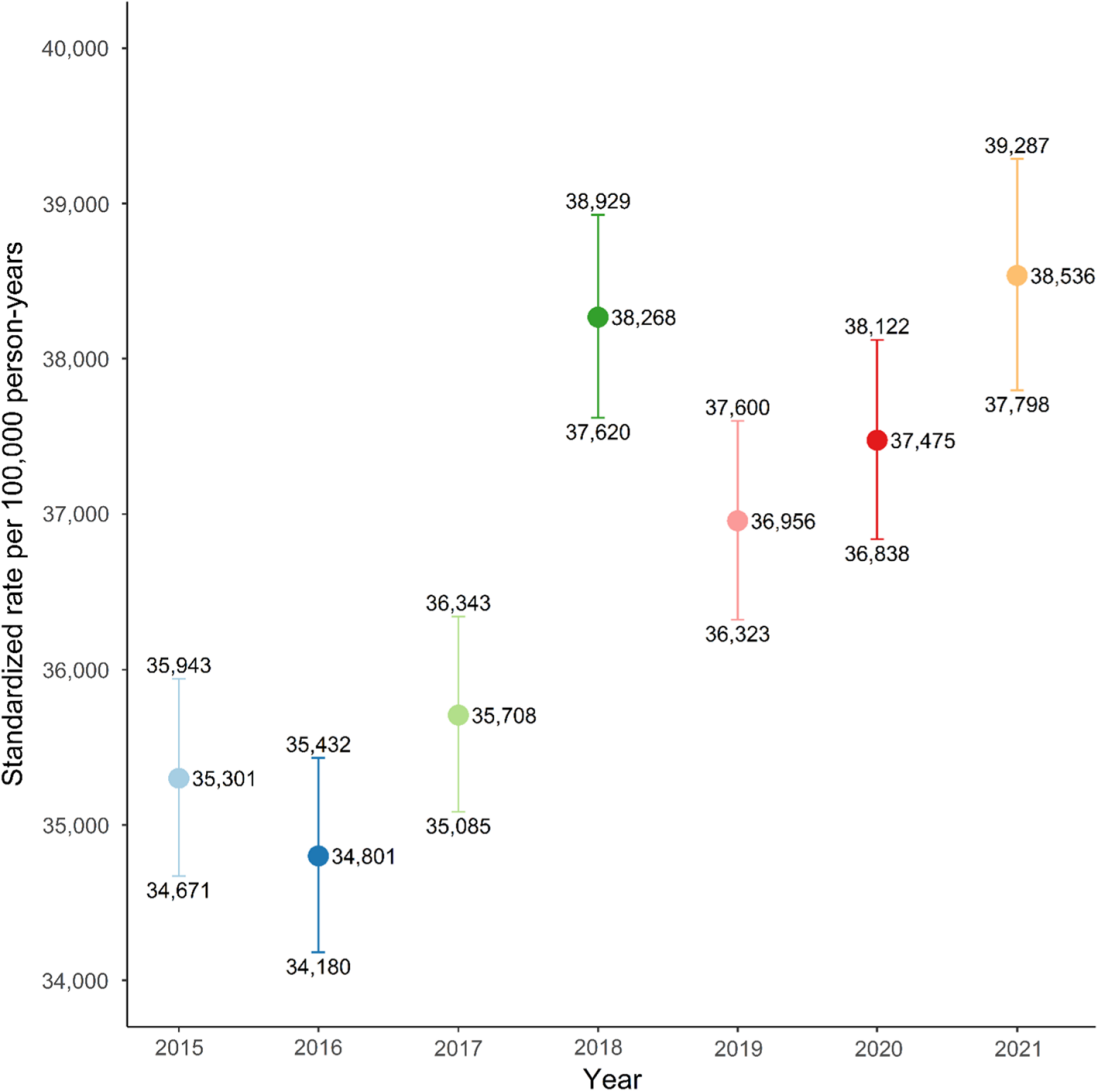
Age- and sex-standardized yearly all-cause mortality rates as number of events per 100,000 person-years for each year from 2015 until October 6, 2021, among 135,501 Danish nursing home residents

The weekly all-cause mortality rates standardized to age and sex reveal similar rates between all years with some weekly variation (Fig. 1). Peaks in mortality were seen in 2018 in week 7 to 15, and again through the weeks 49 to 52 in 2020, and likewise in 2021 in week 1 to 6. The yearly all-cause mortality rates standardized to age and sex for the years 2015, 2016, and 2017 showed similar mortality rates of 35,301 (95% CI: 34,671 to 35,943), 34,801 (95% CI: 34,180 to 35,432), and 35,708 (95% CI: 35,085 to 36,343) per 100,000 person-years, respectively (Fig. 2). These rates were slightly lower compared to the subsequent years. Year 2018 through October 2021 showed similar rates; in 2018 the yearly mortality rate was 38,268 (95% CI: 37,620 to 38,929) per 100,000 person-years. The following years of 2019, 2020, and 2021 had yearly mortality rates of 36,956 (95% CI: 36,323 to 37,600), 37,475 (95% CI: 36,838 to 38,122), and 38,536 (95% CI: 37,798 to 39,287) per 100,000 person-years, respectively (Fig. 2). The sensitivity analysis to test seasonal infectious disease variation showed similar results as the primary outcome (Supplementary Figure S1).

### Secondary outcomes

**Fig. 3:**
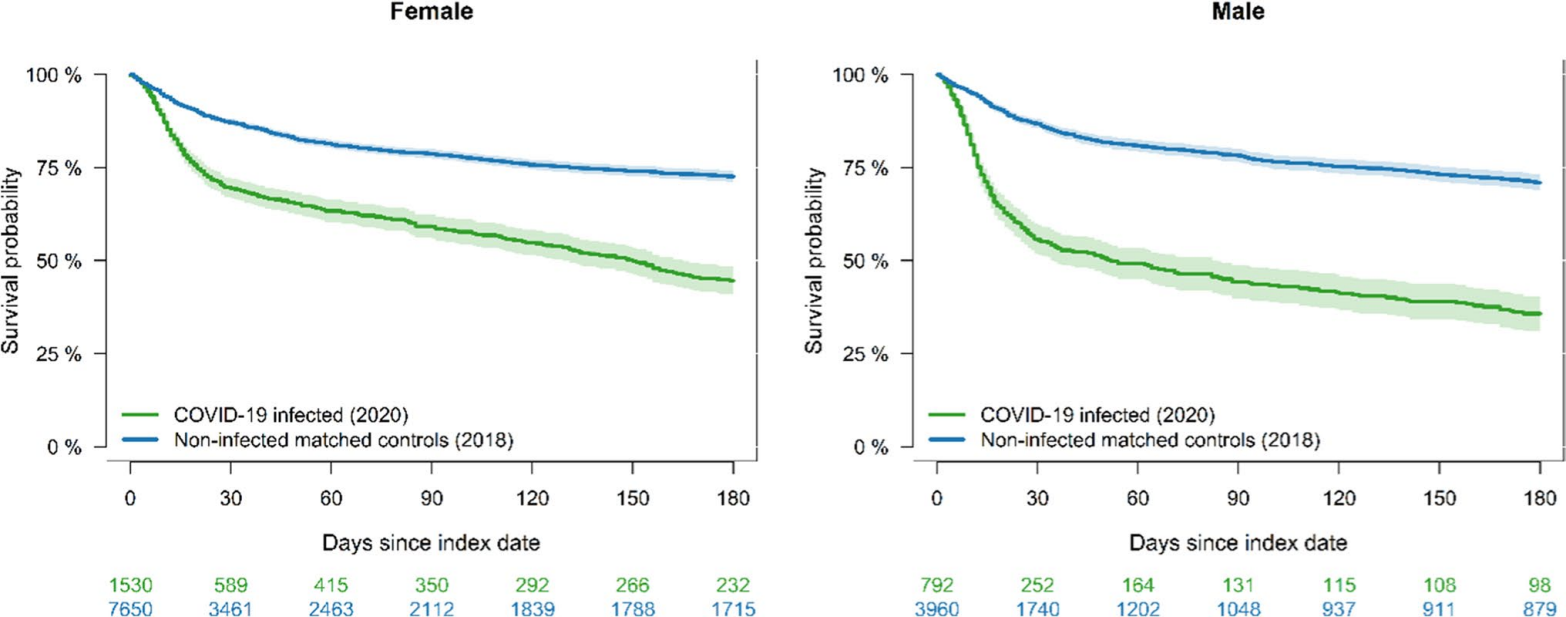
The survival probability within 180 days for COVID-19-infected nursing home residents in 2020 matched with non-infected residents in 2018 and stratified by sex. For SARS-CoV-2-infected males the lifetime lost difference of 56 days (95% CI: 48 to 63), whereas the lifetime lost difference for infected females was 35 days (95% CI: 30 to 40). For both combined, the lifetime lost difference was 42 days (95% CI: 38 to 46)

For both males and females, the survival probability within 180 days was under 50% for COVID-19-infected residents in 2020 compared to a survival probability of almost 75% for the non-infected in 2018 (Fig. 3). Similar results were obtained for the other pre-pandemic years of 2015 to 2017 and 2019 (Supplementary Figures S3-6). Likewise, extending the survival probability to 365 days also showed similar result (Supplementary table S2). SARS-CoV-2-infected males in 2020 had the highest mortality, with a lifetime lost difference of 56 days (95% CI: 48 to 63) compared to their non-infected counterparts in 2018. For infected females in 2020 versus non-infected females in 2018, the lifetime lost difference was 35 days (95% CI: 30 to 40), while for both combined it was 42 days (95% CI: 38 to 46).

**Fig. 4:**
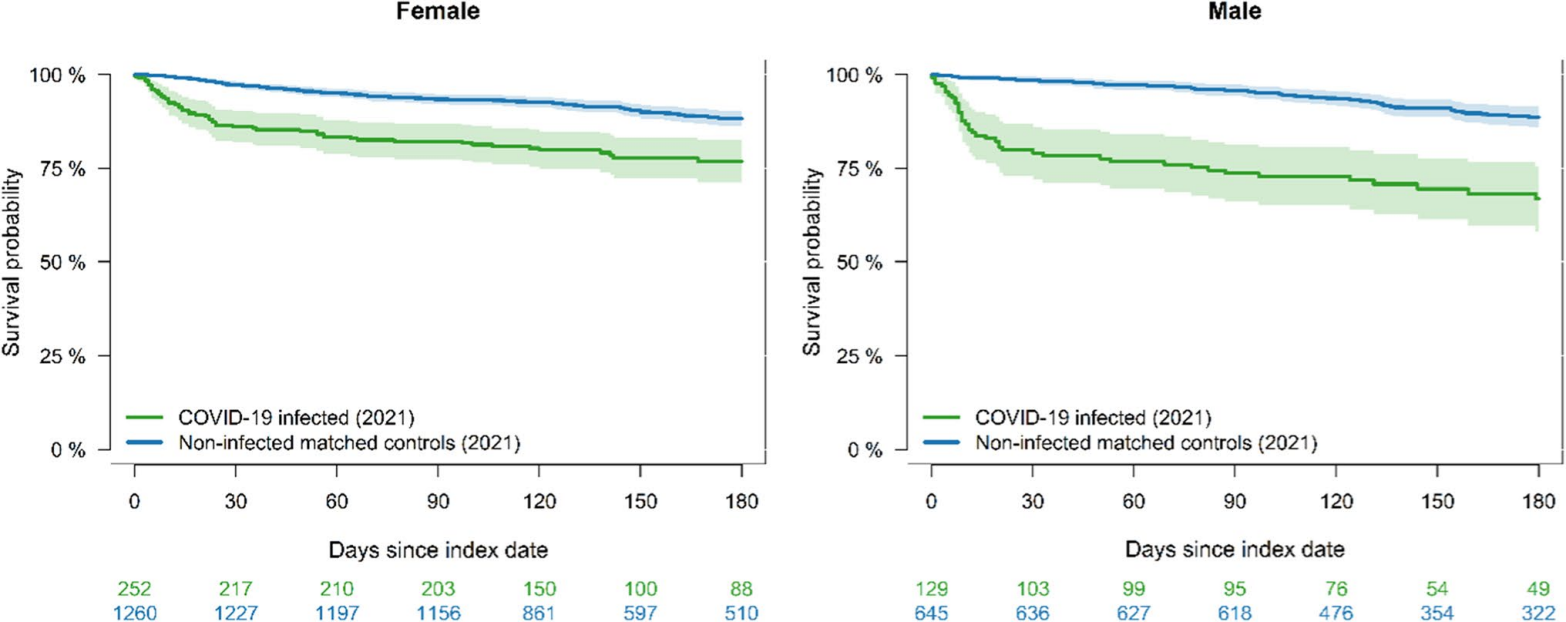
The survival probability within 180 days among vaccinated nursing home residents, matching COVID-19 infected with non-infected counterparts in 2021 and stratified by sex. For SARS-CoV-2-infected males the lifetime lost difference of 35 days (95% CI: 23 to 48), whereas the lifetime lost difference for infected females was 20 days (95% CI: 12 to 28). For both combined, the lifetime lost difference was 25 days (95% CI: 18 to 32)

The survival probability within 180 days among vaccinated nursing home residents, matching SARS-CoV-2-infected with non-infected counterparts in 2021, was almost 80% for SARS-CoV-2-infected residents and above 85% for non-infected, for both males and females (Fig. 4). SARS-CoV-2-infected males had the highest mortality with a lifetime lost difference of 35 days (95% CI: 23 to 48), whereas the lifetime lost difference for infected females was 20 days (95% CI: 12 to 28). For both combined, the lifetime lost difference was 25 days (95% CI: 18 to 32).

## Discussion

The main result from this nationwide study was that even though a high proportion of fatal COVID-19 cases in Denmark occurred among nursing home residents, the annual mortality of nursing home residents during 2018–2021 only had minor variations. However, slightly lower mortality was observed in the non-pandemic years of 2015 through 2017 in comparison to the previously mentioned years. For the individual nursing home resident, SARS-CoV-2-infection increased the risk of death in both vaccinated and unvaccinated residents. During single weeks, peaks in all-cause mortality were observed in both 2020 and 2021, but not more than during the influenza pandemic of 2018, where the influenza vaccine failed to deliver protection [8] without raising major public concern. Although the weekly peaks could imply excess mortality, nursing home residents are more susceptible to sustaining more severe and potentially fatal causes of disease, including influenza and COVID-19 [4, 5, 7]. For instance, the influenza epidemic in the winter of 2018 as well as the COVID-19 pandemic, particularly in the winter of 2020, were likely the main drivers for the mortality peaks seen during these periods. However, across all calendar years no major change in mortality was seen, and the years 2018 until October 2021 only minor differences were seen, supporting the notion of disease susceptibility among frail, elderly nursing home residents. We can only speculate on the minor mortality differences between 2020 and 2021 as the preventive initiatives where almost the same in these years, apart from the initiation of COVID-19-vaccine distribution around the turn of 2020/2021.

The study establishes a negative impact of nursing home residents related to COVID-19. Males had the lowest survival probability with a lifetime lost difference of 56 days, which was not surprising, as a previous study demonstrated that SARS-CoV-2-infected males had a higher risk of all-cause death compared to their female counterparts [24]. Infected females still had a lower survival probability with a lifetime lost difference of 35 days compared to their non-infected counterparts. Similar findings were established among the vaccinated residents in 2021, where males still had the highest mortality with a lifetime lost difference of 35 days, whereas females had a lower lifetime lost difference of 20 days. This supports that SARS-CoV-2-infected males have worse prognosis than females.

Independently of sex, in the comparison between 2020 and the year 2018, the lifetime lost difference was 42 days among infected nursing home residents compared to their non-infected counterpart within 180 days. Among the vaccinated residents in 2021, the study likewise found the lowest survival probability in the SARS-CoV-2-infected residents. Nevertheless, a lifetime lost difference of just 25 days was found for both sexes. In this context, vaccines seem to have a positive impact on reducing the lifetime lost among nursing home residents, rendering it reasonable that nursing homes residents have the highest prioritization for vaccine distribution. Even so, nursing home residents who get infected with SARS-CoV-2 still have lifetime lost and the lowest survival probability. Therefore, implementation of preventive strategies such as isolation and minimization of external contacts seems reasonable as this is well documented to prevent spreading of SARS-CoV-2-infection. However, isolation and deprivation of external contact most likely carry other costs, such as mental disturbances and loneliness among nursing home residents. In this study it was not possible to include data on mental health and quality of life, but in light of negligible impact on overall mortality and relative loss of lifetime, it could be important to study unrevealed effects on mental health and quality of life. Another limitation of this study includes potentially unmeasured confounding, due to the observational nature of the study. However, the current cohort study design includes all Danish nursing home residents with exact background information from administrative nationwide registers and a complete follow-up of the residents. The nationwide data and design minimize the risk of selection bias and thereby increase the generalizability to similar nursing home settings in other countries.

Although, a negative impact of COVID-19 on mortality was established in nursing home residents, it is noteworthy that only minor changes in yearly mortality were observed between the pre-pandemic and the pandemic years. This highlights that nursing home residents simply have a high mortality with a short life expectancy. Healthcare authorities, politicians and decisionmakers should therefore account for life expectancy and yearly mortality among frail and diseased populations in society, such as nursing homes residents, when estimating and reporting numbers of casualties caused by epidemics or pandemics.

In conclusion, nursing home residents infected with SARS-CoV-2 had a substantial mortality and a large proportion of all COVID-19 related deaths in Denmark were among nursing home residents. But the overall mortality among nursing home residents in Denmark was only slightly elevated due to the pandemic which may not be generalizable to other settings and countries. During the continued management of the pandemic and for future epidemics or pandemics it is important to report fatal cases in proper perspective of the overall mortality.

## Supplementary Information

Below is the link to the electronic supplementary material.

Supplementary file1 (DOCX 925 kb)

## Data Availability

The dataset generated during the current study is not publicly available. All data were accessed in the research environment of Statistics Denmark where multiple registries can be combined with the limitation that individual data cannot be exported from the research environment. Further, individual information is encrypted. Thus, datasets cannot be made available. Other researchers that wish to access the data and codes can contact the authors of this study for collaboration on further studies.
