# Supplementary material for: All-cause mortality among Danish nursing home residents before and during the COVID-19 pandemic: a nationwide cohort study
Source: Eur J Epidemiol. 2023 Apr 3;38(5):523–31. doi: 10.1007/s10654-023-00994-6 (PMC10069726; doi:10.1007/s10654-023-00994-6)
Supplement: Supplementary file 1 — Supplementary file1 (DOCX 925 kb) [file 10654_2023_994_MOESM1_ESM.docx]

**Supplementary Information**

Supplementary Table S1 – Conditions and diagnosis codes

| Condition | | Subgroups included | ICD-10 codes | ICD-8 codes |
| --- | --- | --- | --- | --- |
| Chronic airways disease | | Asthma, chronic obstructive pulmonary disease, interstitial lung disease | I278-79, J40-7, J60-7, J68.4, J70.1, J70.3, J84, J96-1, J92.0, J95.3, J96.1, J98.2-3 | 490-3, 515-8 |
| Chronic kidney disease | |  | I12.0, I13.1, N01.8-9, N03.2-7, N05.2-7, N25.0, Z49.0-2, Z94.0, Z99.2 | 403, 404, 581-584, 59009, 59139, 75310-9, 792 |
| Diabetes | |  | E10-14 | 24900-25009 |
| Cardiovascular | Cardiac | Myocardial infarction | I21, I22, I25 | 410 |
|  |  | Heart failure | I09.9, I11.0, I13.0, I13.2, I25.5, I42.5-7, I42.8A, I42.9, I43, I50, P29.0 | 42709-11, 42179, 78249 |
|  | Other circulatory disease | Hypertension | I10-15 | 40009-40199 |
|  |  |  |  | At least two different ATC groups (24):  1) renin-angiotensin inhibitors C09,  2) calcium channel blockers: C07F, C08, C09BB, C09DB; 3) non-loop diuretics: C02DA, C02L, C03A, C03B, C03D, C03E, C03X, C07C, C07D, C08G, C09BA, C09DA, C09XA52),  3) vasodilators: C02DB, C02DD, C02DG, C04, C05,  4) β blockers: C07,  5) α adrenergic blockers: C02A, C02B, C02C |
|  |  | Cerebrovascular disease | I60-69, G45, G56, H24.0 | 430-438 |
|  |  | Peripheral vascular disease | I70-72, I73.1, I73.8, I73.9, I77, I79.0, I79.2, K55.1, K55.8, K55.9, Z95.8-9 | 440-445 |
| Dementia | | Dementia, Alzheimers diseases | F00, F01 F020, F039, G30, G318B, G318E, G319, G31B, | 290 |
| Cancer (last diagnosis code ≤10 years ago) | | Cancer, metastases, lymphoma, leukaemia | C00-43, C45-97 | 140-172, 174-207, 27559 |

Fig S1 – Age- and sex-standardized weekly all-cause mortality rates as number of events per 100,000 person-weeks starting in week 40 until week 39 the year after, among 135,501 Danish nursing home residents


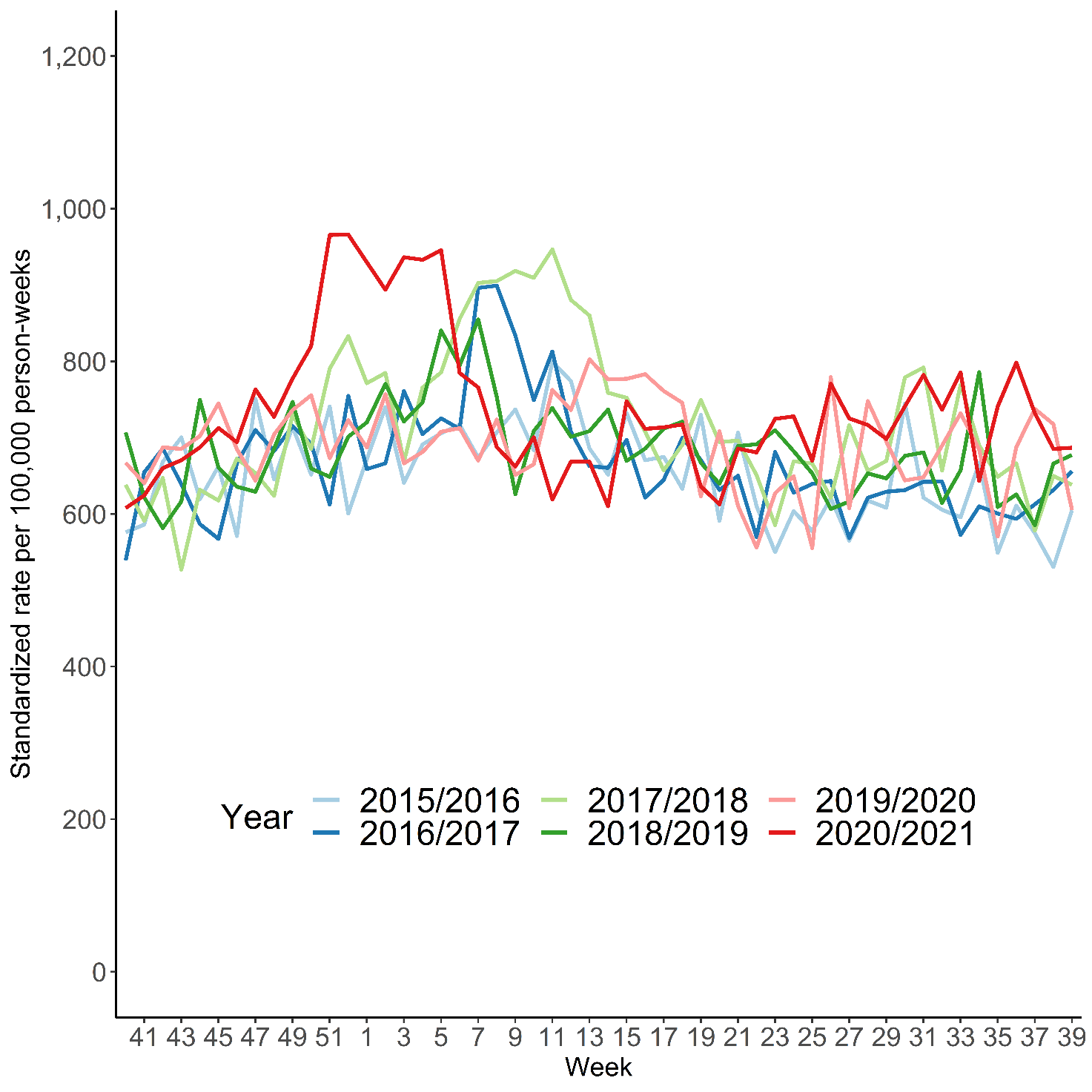


Fig S2 – Age- and sex-standardized weekly all-cause mortality rates as number of events per 100,000 person-weeks starting on January 1 for each year from 2015 until October 6, 2021, with a 4-week running average for each year, among 135,501 Danish nursing home residents.


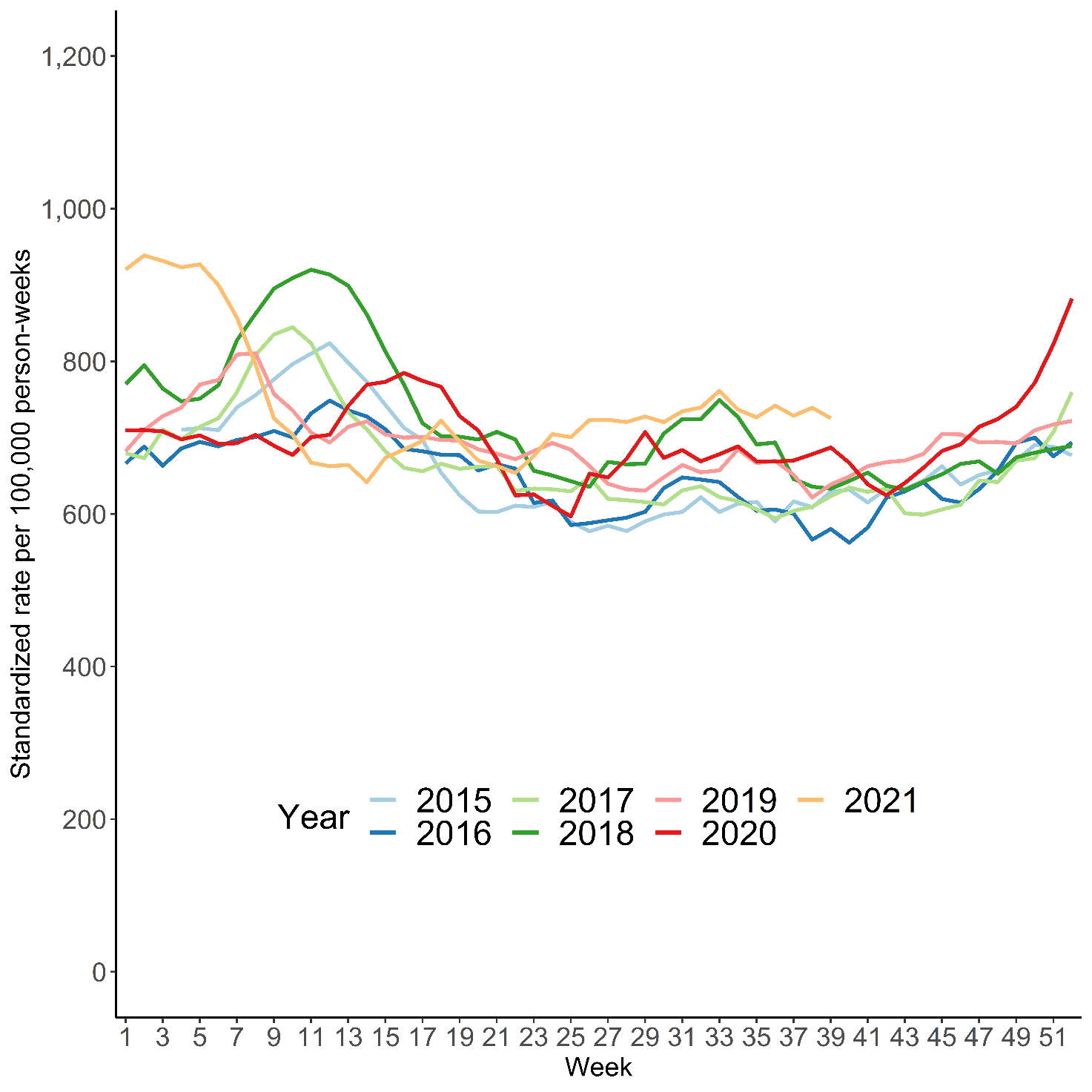


Fig S3 – displays the survival probability within 180 days for COVID-19-infected nursing home residents in 2020 matched with non-infected residents in 2015 and stratified by sex. For SARS-CoV-2-infected males the lifetime lost difference of 55 days (95% CI: 47 to 62), whereas the lifetime lost difference for infected females was 37 days (95% CI: 32 to 42). For both combined, the lifetime lost difference was 43 days (95% CI: 39 to 47).


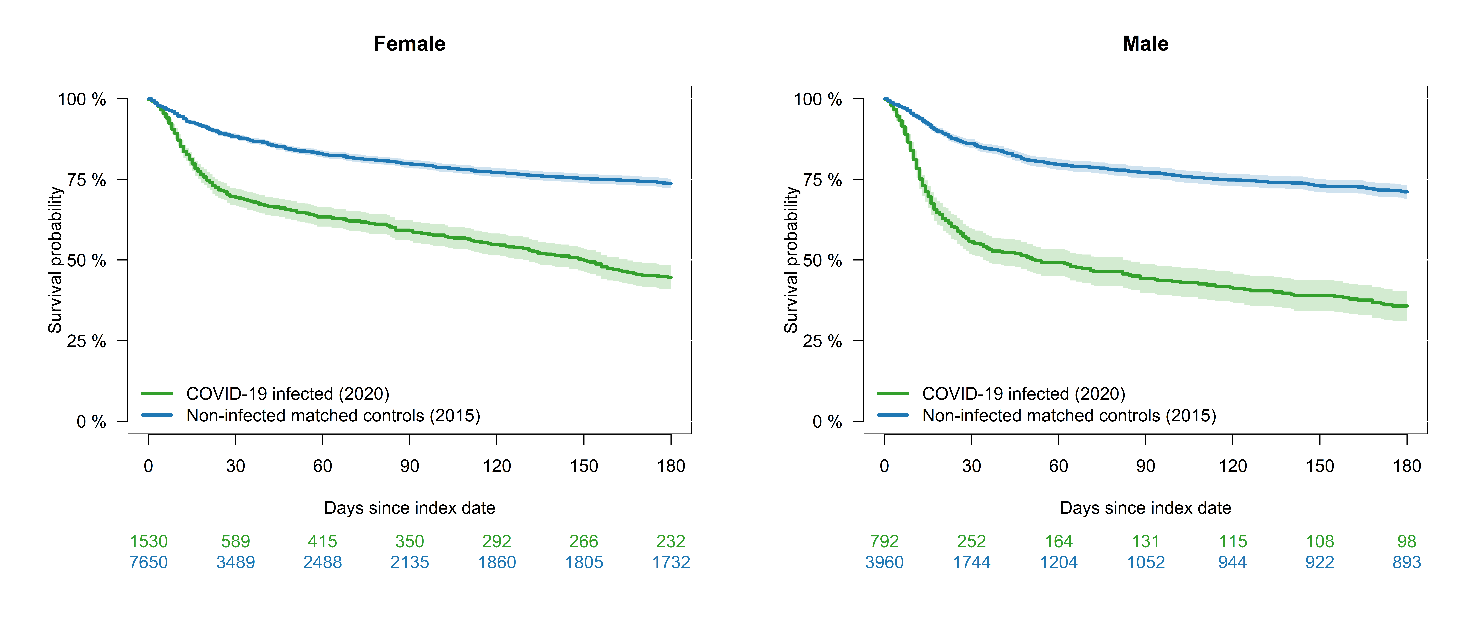


Fig S4 – displays the survival probability within 180 days for COVID-19-infected nursing home residents in 2020 matched with non-infected residents in 2016 and stratified by sex. For SARS-CoV-2-infected males the lifetime lost difference of 54 days (95% CI: 47 to 62), whereas the lifetime lost difference for infected females was 35 days (95% CI: 30 to 40). For both combined, the lifetime lost difference was 42 days (95% CI: 38 to 46).


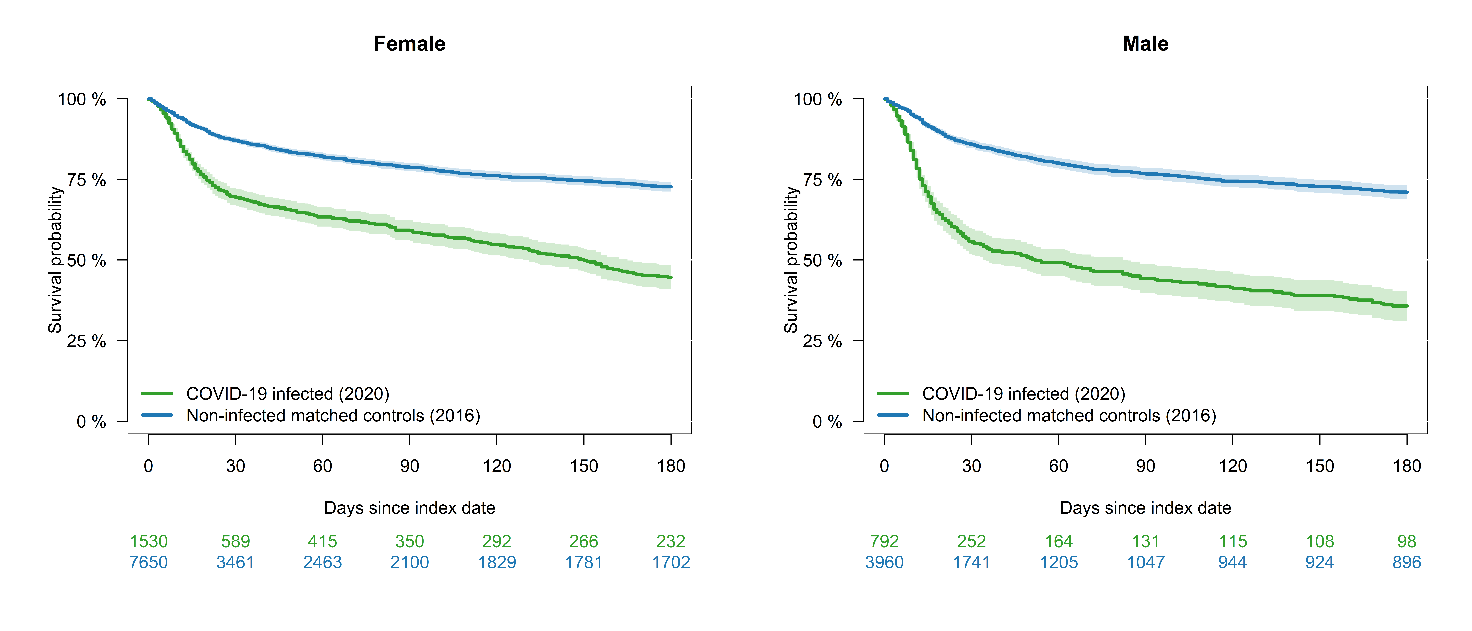


Fig S5 – displays the survival probability within 180 days for COVID-19-infected nursing home residents in 2020 matched with non-infected residents in 2017 and stratified by sex. For SARS-CoV-2-infected males the lifetime lost difference of 49 days (95% CI: 41 to 56), whereas the lifetime lost difference for infected females was 34 days (95% CI: 29 to 39). For both combined, the lifetime lost difference was 39 days (95% CI: 35 to 43).


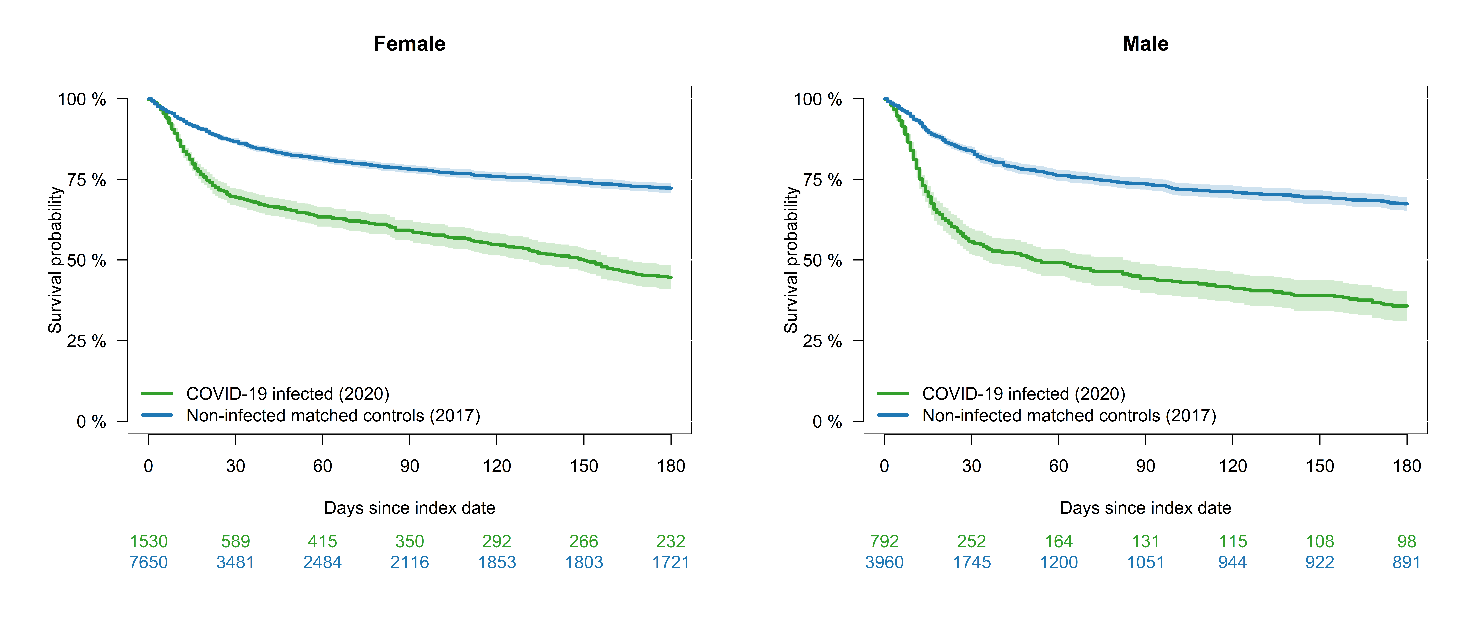


Fig S6 – displays the survival probability within 180 days for COVID-19-infected nursing home residents in 2020 matched with non-infected residents in 2019 and stratified by sex. For SARS-CoV-2-infected males the lifetime lost difference of 56 days (95% CI: 49 to 63), whereas the lifetime lost difference for infected females was 35 days (95% CI: 30 to 40). For both combined, the lifetime lost difference was 42 days (95% CI: 38 to 46).


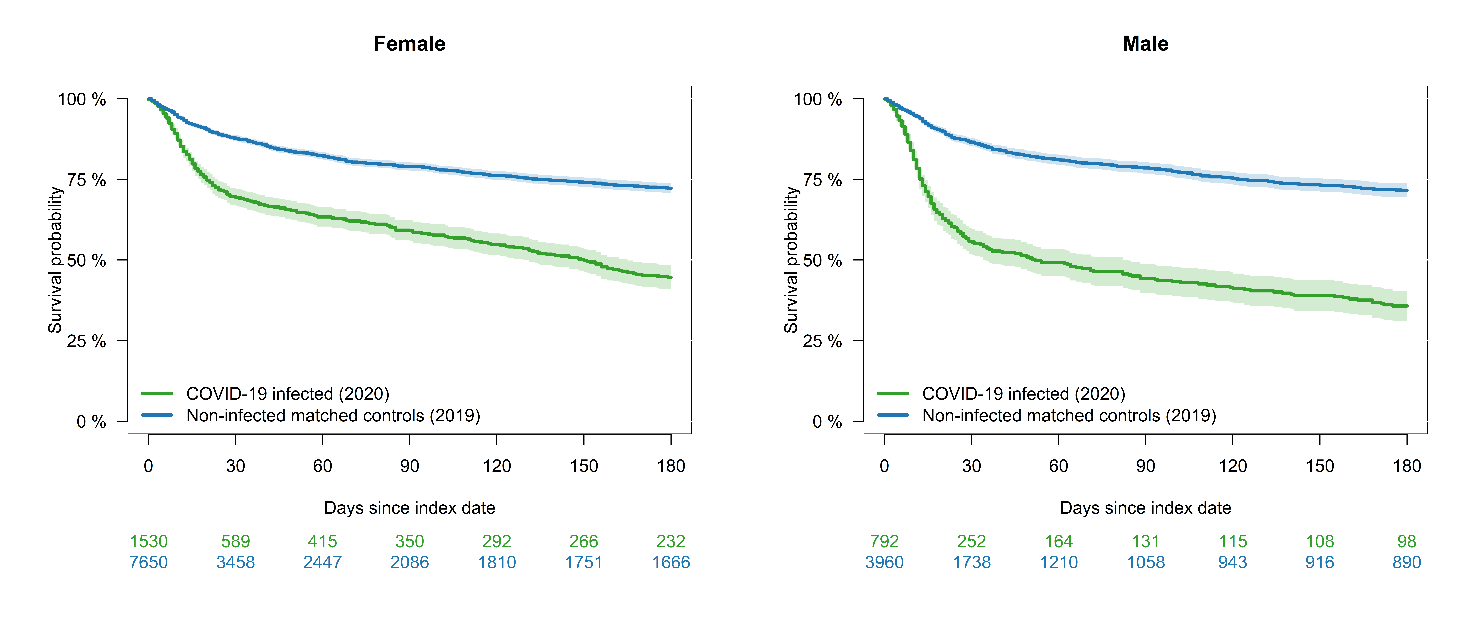


Supplementary Table S2 – The survival probability within 365 days for COVID-19-infected nursing home residents matched with non-infected residents and stratified by sex for years included in the secondary outcomes.

|  | Lifetime lost difference in days | | |
| --- | --- | --- | --- |
|  | Males | Females | Both sexes combined |
| COVID-19-infected nursing home residents in 2020 matched with non-infected residents in 2018 | 57 days (95% CI: 45 to 69) | 31 days (95% CI: 22 to 40) | 40 days (95% CI: 33 to 47) |
| Vaccinated nursing home residents, matching COVID-19 infected with non-infected counterparts in 2021 | 71 days (95% CI: 46 to 98) | 35 days (95% CI: 17 to 52) | 47 days (95% CI: 33 to 62) |
| COVID-19-infected nursing home residents in 2020 matched with non-infected residents in 2015 | 59 days (95% CI: 48 to 72) | 36 days (95% CI: 27 to 44) | 44 days (95% CI: 37 to 50) |
| COVID-19-infected nursing home residents in 2020 matched with non-infected residents in 2016 | 59 days (95% CI: 47 to 72) | 32 days (95% CI: 23 to 41) | 41 days (95% CI: 34 to 48) |
| COVID-19-infected nursing home residents in 2020 matched with non-infected residents in 2017 | 46 days (95% CI: 35 to 58) | 31 days (95% CI: 22 to 40) | 36 days (95% CI: 29 to 43) |
| COVID-19-infected nursing home residents in 2020 matched with non-infected residents in 2019 | 57 days (95% CI: 45 to 69) | 30 days (95% CI: 21 to 39) | 39 days (95% CI: 32 to 46) |
| 95% confidence intervals [95% CI] | | | |
